# Activated Charcoal—A Potential Material in Glucoamylase Recovery

**DOI:** 10.4061/2011/483943

**Published:** 2011-12-14

**Authors:** S. O. Kareem, I. Akpan, T. O. S. Popoola, L. O. Sanni

**Affiliations:** ^1^Department of Microbiology, University of Agriculture, PMB 2240, Abeokuta, Nigeria; ^2^Department of Food Science and Technology, University of Agriculture, PMB 2240, Abeokuta, Nigeria

## Abstract

The potential of activated charcoal in the purification of fungal glucoamylase was investigated. Various concentrations of activated charcoal (1–4% w/v) were used to concentrate crude glucoamylase from *Rhizopus oligosporus* at different temperature values (30–50°C). Effects of pH (3.0–6.0) and contact time (0–60 min) on enzyme purification were also monitored. Activated charcoal (3% w/v) gave a 16-fold purification in a single-step purification at 50°C for 20 min and pH 5.5. The result of SDS-PAGE analysis of purified glucoamylase showed two major protein bands with corresponding molecular weight of 36 kDa and 50 kDa. The method is inexpensive, rapid, and simple which could facilitate downstream processing of industrial enzyme.

## 1. Introduction

Enzyme purification is a necessary prerequisite for a full understanding of the nature and mechanism of action of the enzyme [1]. This is usually carried out by a multistep process involving biomass separation, concentration, primary isolation, purification, and polishing as the main unit operations [2]. The conventional methods for the removal of colloidal particles and enzymatic impurities from fermentation broth using ammonium sulphate precipitation may require extensive dialysis for about 12–16 hour for product recovery and often cause protein denaturation due to conformational changes [3]. The use of carbowax or polyvinyl alcohol for protein and enzyme concentration is also limited by poor water absorbing capacity [4]. Similarly, the use of carboxy-methyl cellulose, tannic acid, and edible gum as precipitants and as well as organic solvents also poses the problem of product recovery [5, 6]. Gel filtration technique is also considered laborious and expensive in the developing countries [6, 7].

 Activated charcoal is an adsorbent widely used in the treatment of wastewater and industrial contaminants by virtue of its high removal capacity and adaptability for a wide range of pollutants [8]. It is made from any essentially carbonaceous materials. Tree bark, coal, cotton waste, palm kernel shell, and many agricultural by- products can be used to produce activated carbon and their ability to remove colours have been reported [9].

 Activated charcoal is used to remove compounds that cause objectionable taste, colour, and odour in water treatment while its industrial applications involve removal of toxic gases and pesticides and as well as purification of organic compounds [10, 11]. It is known that around 80% of the world production of activated charcoal is used in aqueous-phase adsorption of both organic and inorganic compounds [12, 13]. Although application of activated charcoal in the decolorization of enzyme-converted glucose syrup had been reported, its uses in the purification of microbial enzymes have been scanty. This study reports application of activated charcoal in the recovery of fungal amylases.

## 2. Materials and Methods

### 2.1. Microorganism

Amylolytic strains of *Rhizopus oligosporus* were obtained from the culture collection center, Department of Microbiology, University of Agriculture, Abeokuta, Nigeria. It was maintained on potato dextrose agar (PDA) slants at 4°C and subcultured bimonthly.

### 2.2. Chemicals

Activated charcoal, 3, 5-dinitrosalicylic acid and polyacrylamide were from Sigma. All chemicals were reagent grade.

### 2.3. Amylase Production

Rice bran solid-state medium was prepared and inoculated with spores of *Rhizopus oligosporus* according to the method of Akpan et al. [14]. Incubation was at 30°C for 72 h. The crude amylase was recovered by mixing the moldy bran. Moldy bran was mixed with acetate buffer (0.2 M) (pH 4.5) in the ratio 1 : 4 (w/v) in a conical flask. The mixture was shaken on an orbital shaker at 150 rpm at 28°C for 1 h. The extract was then filtered using muslin cloth. The filtrate was used as the crude amylase and kept at 4°C.

### 2.4. Determination of Amylase Activity

The amylase activity was determined by mixing crude amylase with 4% w/v gelatinized cassava starch (pH 4.5) and incubated at 60°C for 1 h. Reducing sugar was determined using dinitrosalicylic acid method of Miller [15]. Protein concentration was estimated using biuret method as described by Koch and Putnam [16].

### 2.5. Purification of Crude Amylase with Activated Charcoal

Studies were carried out on the purification of glucoamylase using activated charcoal (0.8 mm). Various concentrations of activated charcoal (1–4% w/v) were added to crude glucoamylase (pH 4.5) and incubated at 30°C for 30 mins with occasional stirring. The mixture was then centrifuged at 2500 rpm in a bench centrifuge for 10 min. The effect of temperature on enzyme purification capacity of activated charcoal was evaluated at different temperature values (30–60°C). Effect of pH on the purification of glucoamylase was also determined at different pH values (3.5–6.0).

### 2.6. Electrophoresis

Molecular weight of the purified enzyme was estimated using Sodium dodecyl sulphate (SDS) polyacrylamide gel electrophoresis as described by Laemmli [17]. The gels were stained with Coomassie Brilliant blue R-250 (BioRad, USA). The protein bands were estimated and compared with standard protein markers (BioRad, USA).

## 3. Results and Discussion

### 3.1. Effect of Concentration of Charcoal

**Figure 1:**
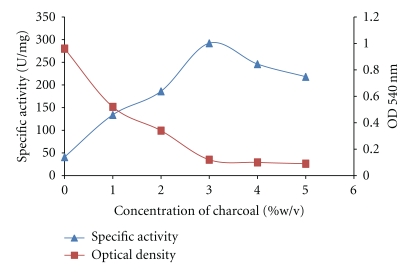
Effect of concentration of activated charcoal on purification of glucoamylase.

The result presented in Figure 1 showed the effect of various concentrations of activated charcoal on the purification of fungal amylase. A significant increase was noted in the specific activity of the glucoamylase as the concentration of charcoal increases up to 3% w/v with the optimum-specific activity of 250 U/mg. A further increase beyond 3% (w/v) led to a decrease in their specific activity. About 90% reduction in turbidity was observed in the treated sample. It was observed that the rate of absorption changed in magnitude with the increase in the amount of charcoal used. The degree of absorption has been reported to be influenced by accessibility of the adsorbate to the adsorbent [18].

### 3.2. Effect of Temperature on Amylase Purification

**Figure 2:**
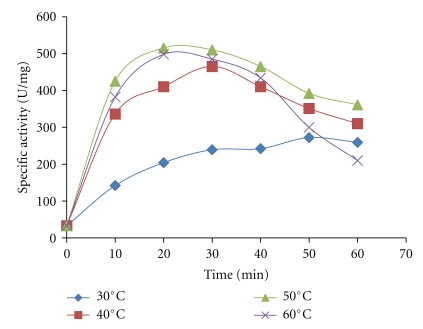
Effect of temperature on purification of glucoamylase using activated charcoal (3% w/v).

The result presented in Figure 2 summarized the effect of time and temperature on the purification of amylase using activated charcoal. It was evident that purification of fungal amylase by charcoal at elevated temperatures gave a marked increase in the specific activity of fungal amylase. At 50°C, optimum specific activity of 520 U/mg was attained at 20 min resident time. However, enzyme treatment with charcoal at temperature beyond 50°C and at a longer period beyond 30 min resulted in a decrease in amylase activity. It is evident that elevated temperature enhanced protein absorption and decolorizing capacity of activated charcoal. The rate and capacity of absorption depends mainly on the surface chemistry of activated charcoal, contact time, and temperature [8, 19].

### 3.3. Effect of pH on Glucoamylase Purification

Table 1 showed the effect of pH on the purification of fungal glucoamylase by activated charcoal. It was observed that pH values between 4.5 and 5.5 enhanced the specific activity of the fungal amylase with optimum purification index at pH 5.5. However, at pH ≥5.5 there was a decrease in the specific activity of the enzyme. This paper conforms with optimum pH range for amylases [20]. Ability to purify the amylase at pH 4.0–5.5 which falls within the optimum pH values for amylase is also an added advantage.

### 3.4. Purification of Glucoamylase under Optimized Conditions

Purification profile of glucoamylase with charcoal under optimized conditions gave a 16-fold purification in a single step (Table 2). The result is a marked improvement over the conventional method of enzyme purification such as ammonium sulphate precipitation. Interestingly, the glucoamylase was left in the supernatant and the effectiveness of activated charcoal at low concentration had been noted as an added advantage at pH 4.5. The fact that enzyme does not precipitate with activated charcoal may be due to the selective absorption of proteins which is attributed to fine network of pores, distribution of pore sizes, the type of functional group on the surface, contact time, and temperature [8, 19].

**Figure 3:**
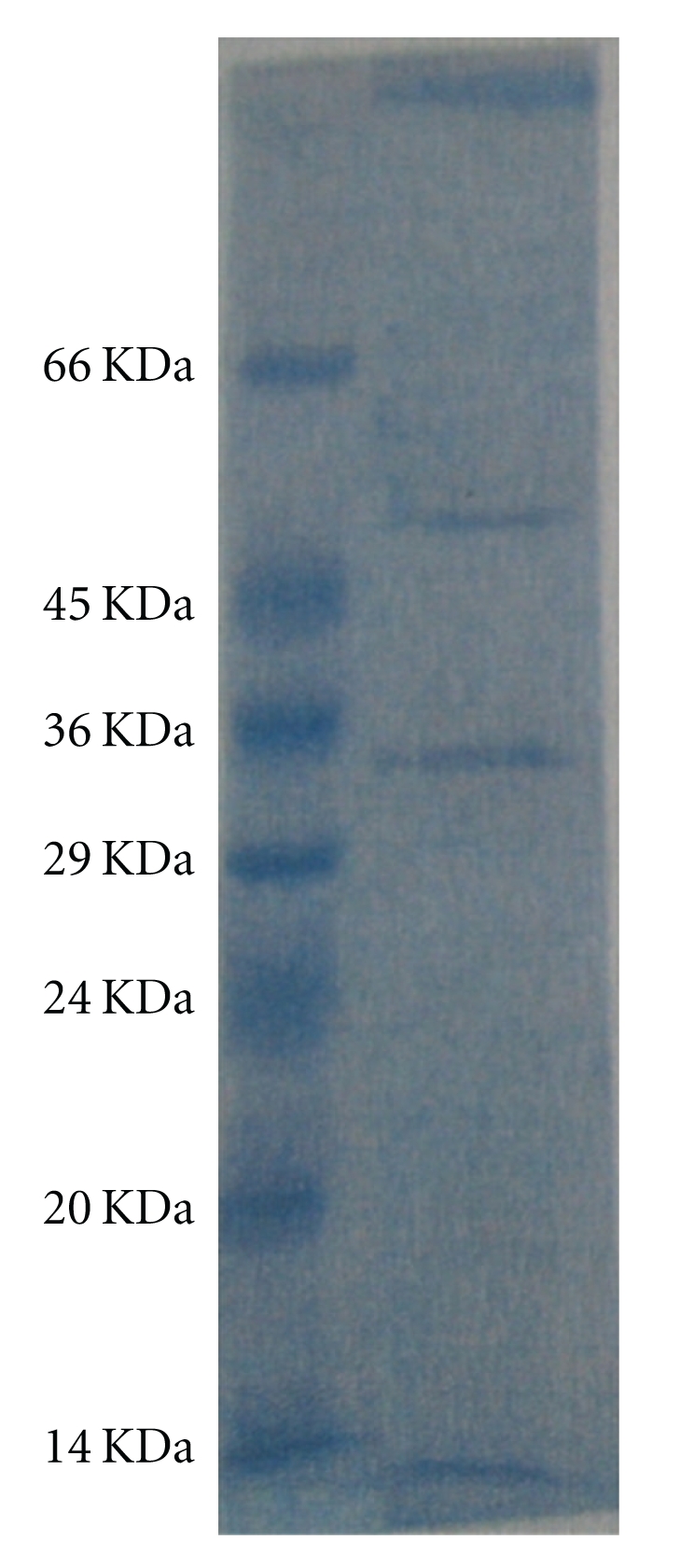
Molecular weight of purified glucoamylase on SDS-PAGE gel.

Rapid purification of enzyme from a complex fermentation broth mixture at a high purification fold put activated charcoal at an advantage over conventional purification techniques. The conventional methods of enzyme purification including salting out technique, solvent precipitation, and gel filtration are not always economical from an industrial stand point because, they are associated with some problems particularly such as difficulty of scaling up and plugging when treating crude extracts which often contain viscous and particulate materials [18, 21]. Furthermore, recovery or disposal of materials used in the separation process may increase the cost of the separation step, and therefore, it is expensive for developing economy [19]. Therefore, the use of activated charcoal is considered as alternative method of enzyme purification. The result of SDS-PAGE analysis of purified glucoamylase (Plate 1) showed two major protein bands with corresponding molecular weight of 36 kDa and 50 kDa which falls within apparent molecular weights for fungal glucoamylases (see Figure 3) [18].

 Efficient surface absorption characteristics in addition to the low cost of activated charcoal can be harnessed for depolarization of fermented medium for effective recovery and purification of industrial enzymes which can make the downstream processing in large-scale industrial bioprocesses more cost effective [19, 21]. The result confirms activated charcoal as a good clarifying agent and unveils its potential as material for enzyme concentration.

## 4. Conclusion

In this study, glucoamylase was recovered from the fermentation broth by activated charcoal at 50°C for 20 minutes. After the elution process, a highly concentrated and purified glucoamylase was obtained in less than 30 minutes. This technique looks promising, cheap, and rapid in downstream processing of industrial enzymes.

## Figures and Tables

**Table 1:** Effect of pH on purification of glucoamylase by activated charcoal.

pH	Amylase activity U/mL	Protein conc. mg/mL	Specific activity U/mg
3.0	1430	14.16	101
3.5	1478	9.60	154
4.0	1598	8.83	181
4.5	1675	3.57	469
5.0	1684	3.20	526
5.5	1686	3.38	496
6.0	1640	3.87	424

**Table 2:** Summary of purification of glucoamylase.

Steps	Amylase activity U/mL	Protein mg/mL	Specific activity U/mg	Purification fold	Yield %
Crude amylase	1702	50	33	—	100
Activated charcoal	1684	3.2	526	16	81
